# Health and zoonotic Infections of snow leopards *Panthera unica* in the South Gobi desert of Mongolia

**DOI:** 10.1080/20008686.2019.1604063

**Published:** 2019-06-05

**Authors:** Carol Esson, Lee F. Skerratt, Lee Berger, Jonas Malmsten, Tanja Strand, Åke Lundkvist, Josef D. Järhult, Johan Michaux, Tserennadmid Nadia Mijiddorj, Rana Bayrakçısmith, Charudutt Mishra, Örjan Johansson

**Affiliations:** a One Health Research Group, College of Public Health, Medical and Veterinary Sciences, James Cook University, Townsville, Australia; b Melbourne Veterinary School, University of Melbourne, Parkville, Australia; c Department of Pathology and Wildlife Diseases, National Veterinary Institute, Uppsala, Sweden; d Department of Wildlife, Fish and Environment Science, Swedish University of Agricultural Sciences, Umeå, Sweden; e Zoonosis Science Centre (ZSC), Department of Medical Biochemistry and Microbiology, Uppsala University, Uppsala, Sweden; f Zoonosis Science Centre (ZSC), Department of Medical Sciences, Uppsala University, Uppsala, Sweden; g Génétique de la conservation Life Sciences, Liege, Belgium; h Snowleopard Conservation Foundation Ulaanbaatar, Mongolia; i Panthera, New York, NY, USA; j Nature Conservation Foundation, Mysore, India; k Grimsö Wildlife Research Station, Department of Ecology, Swedish University of Agricultural Sciences, Riddarhyttan, Sweden; l Snow Leopard Trust, Seattle, WA, USA

**Keywords:** Snow leopard, zoonoses, conservation, one health, Mongolia, ticks

## Abstract

**Background**: Snow leopards, *Panthera uncia*, are a threatened apex predator, scattered across the mountains of Central and South Asia. Disease threats to wild snow leopards have not been investigated.**Methods and Results**: Between 2008 and 2015, twenty snow leopards in the South Gobi desert of Mongolia were captured and immobilised for health screening and radio-collaring. Blood samples and external parasites were collected for pathogen analyses using enzyme-linked immunosorbent assay (ELISA), microscopic agglutination test (MAT), and next-generation sequencing (NGS) techniques. The animals showed no clinical signs of disease, however, serum antibodies to significant zoonotic pathogens were detected. These pathogens included, *Coxiella burnetii*, (25% prevalence), *Leptospira* spp., (20%), and *Toxoplasma gondii* (20%). Ticks collected from snow leopards contained potentially zoonotic bacteria from the genera *Bacillus, Bacteroides, Campylobacter, Coxiella, Rickettsia, Staphylococcus* and *Streptococcus*.**Conclusions**: The zoonotic pathogens identified in this study, in the short-term did not appear to cause illness in the snow leopards, but have caused illness in other wild felids. Therefore, surveillance for pathogens should be implemented to monitor for potential longer- term disease impacts on this snow leopard population.

## Introduction

While the overall decrease in biodiversity is often attributed to environmental changes such as land clearing, habitat d estruction, feral pests and climate change, emerging infectious disease can also act as a primary or contributory cause [1–3]. Pathogens can particularly impact endangered and threatened species where populations are already depleted and genetic diversity may be low [4]. The rarity of endangered species makes them challenging to sample systematically, but constant surveillance and collection of baseline health data with ongoing monitoring will aid in determining the impacts of disease in threatened species.

Snow leopards (*Panthera uncia*) are a rare and threatened species, occurring in the high mountains of South and Central Asia including the Himalayas in the south, through the Pamirs, Tien Shan and Altay in the north. The population of reproductive snow leopards is believed to be fewer than 4500 and is continuing to decline [5,6]. In 2008, the Snow Leopard Trust and Snow Leopard Conservation Fund initiated an ongoing, long-term ecological study of snow leopards in the South Gobi Province of Mongolia. During 2011 the field team recorded four snow leopard carcasses on separate locations, three within the study area in the Tost Mountains and one in the Gurvan Saikhan Mountain range, an adjacent range to the northeast. The causes of mortality were not established. There were no signs of trauma or starvation therefore other potential causes of death included infectious diseases. Two of the dead snow leopards were radio-collared territorial males with no overlap in their home ranges, suggesting independent causes of death. The finding of these dead snow leopards prompted the initiation of this disease study.

Despite the range of investigations into threats to snow leopards, none have addressed the prevalence or impacts of disease in the wild. All prior reports on diseases in snow leopards are restricted to zoo animals and include common feline viruses such as feline parvovirus, calicivirus, feline infectious peritonitis (a viral disease caused by a strain of feline coronavirus), feline immune deficiency virus and canine distemper virus, and a papillomavirus specific to snow leopards [7,8]. Non-viral diseases have included veno-occlusive disease, ocular colobomas, tumours and Tyzzer’s disease [9–12]. Several zoonotic diseases such as, leptospirosis, tuberculosis and *Toxascaris* infection have also been recorded [13,14].

Threatened species are not likely to support the circulation of species- specific infectious agents because densities of threatened species are generally low and intra-species interactions are in many cases infrequent [15]. As snow leopard numbers are low, detecting pathogens specific to felids and determining their effects would be challenging and limited. However, pathogens with several species of reservoir hosts, as in the case of zoonotic pathogens, may be readily detected and can impact species at low abundance through disease spill-over [16].

The most prevalent zoonotic infections reported in Central Asian Mountain livestock are rabies, anthrax, plague, leptospirosis, Q fever, brucellosis, toxoplasmosis and echinococcosis [17–19]. Endemic zoonoses are often under-reported due to a lack of public awareness and public health services and so the disease threat may be greater than what is reflected in the literature [20]. Snow leopards live alongside nomadic herders and their livestock throughout their range [21], which may be sources of infection to snow leopards and vice versa.

This study aimed to investigate important zoonotic pathogens that may impact the conservation of snow leopards in Mongolia. Due to the low numbers of snow leopards available for sampling, combined with the possibility of other species endemic to the area that could act as reservoir hosts for pathogens combined with the closeness of the nomadic herders to all components of their environment, we decided to target zoonotic pathogens that can circulate between different host species and hence also impact the health of snow leopards. The zoonotic pathogens selected to sample for were based on prior occurrence in Mongolia, pathogenicity, the potential to infect snow leopards and potential economic losses for the herders. These pathogens included *Coxiella burnetii, Toxoplasma gondii* and *Leptospira* spp. Two of the most severe zoonoses, anthrax and rabies, are known to occur within the study area but were not tested for as we were looking at prior exposure and not active infection. The potential of identifying positive results for those two pathogens would have been highly unlikely because of their extreme pathogenicity [22,23].

## Methods

### Study area

**Figure 1.**
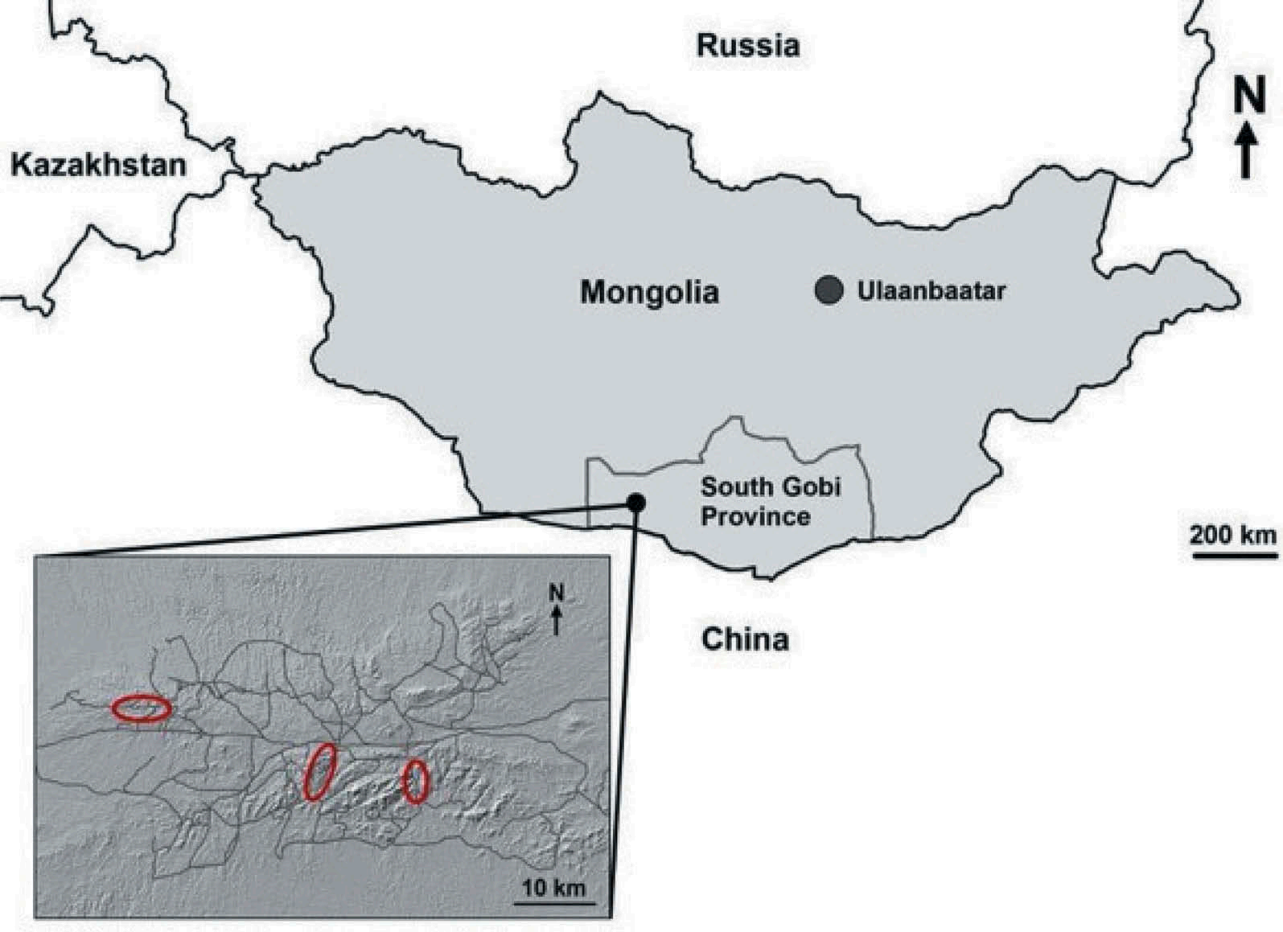
Location of the study area in the South Gobi Desert of Mongolia. Insert shows the topography of the region with trapping areas delineated in red. Grey lines are small roads and tracks that traverse the area [27].

This study was conducted in the Tost Mountains (43° N, 100° E) in the Gobi Desert in southern Mongolia from 2012 to 2015 (Figure 1). The Tost Mountains cover an area of approximately 1700 km^2^ and the population of snow leopards, estimated annually, was between 10–14 adults during our study [24]. The area is also home to approximately 90 herder families, their goats (*Capra aegagrus hircus*), sheep (*Ovis aries*), horses (*Equus caballus*), camels (*Camelus bactrianus*) and domestic dogs (*Canis lupus familiaris*). Other wild species in the area include the grey wolf (*Canis lupus*), corsac fox (*Vulpes corsac*), red fox (*Vulpes vulpes*), Pallas cat (*Otocolobus manul*), Eurasian lynx (*Lynx lynx*), Eurasian wildcat (*Felis silvestris*) and several members of the mustelid family [24,25]. Ungulates include the Siberian ibex (*Capra sibirica*) and Argali sheep (*Ovis ammon*), which are a major component of the snow leopard’s diet, along with livestock [26].

### Handling and measurements

Twenty snow leopards were captured and sampled in conjunction with an ongoing radio telemetry study (see [27] for detailed capture methods). Captures were performed under permits from The Mongolian Ministry of Environment and Green Development. Snow leopards were darted and chemically immobilised with a combination of medetomidine and tiletamine-zolazepam with a mean dose rate of 0.02 ± 0.004 mg/kg body mass medetomidine and 2.17 ± 0.45 mg/kg tiletamine-zolazepam [27].

Sedation lasted approximately one hour, which permitted fitting of a radio-collar for monitoring snow leopards movements for 12 to 18 months, physical examination and collection of blood samples and external parasites. Atipamezole hydrochloride (Antisedan vet 5 mg/mL, Orion Pharma Animal Health, Espoo, Finland) reverses the effects of medetomidine and was administered intramuscularly to aid a smooth recovery from the sedation when handling was completed [27]. Thorough clinical examinations ascertained the general health and body condition of the snow leopards as outlined below.

Due to lack of a standard method for measuring body condition in snow leopards and limited descriptions for other wild felids [28] we developed a consistent field technique for measuring body condition. This technique was based on the amount of muscle over the shoulders and hips and whether the outline of ribs, scapular spine and iliac crest were visible and could be palpated. If scapular spine and iliac crest of hip bones were prominent, then the body condition was ‘poor‘; if bony prominences were difficult to palpate the body condition was rated ‘good‘. In between these two extremes, body condition was scored as ‘moderate’. Pelage was examined for thickness, signs of rubbing, alopecia and wounds. Eyes were examined for any sign of injury or defects. The oral cavity, including tongue and gingiva, were inspected for inflammation, ulcers or other lesions. Teeth were also examined as one of the indicators of age, or to see if broken or infected. Age estimation was based on body mass and tooth wear and tooth colouration [27]. The animals develop a darker cream tooth colour as they age. Scarring of the face in males was also used as an approximate indicator of age as younger males (<two-three years) have few or no scars [27]. Presumably older males obtain facial scars from territorial or resource fights. Females that had not apparently reproduced (< three years of age) typically had nipples that were lighter in colour and smaller than those of known reproductive females.

Body weight was recorded and heart rate, respiratory rate, body temperature and oxygen saturation was measured at 10-minute intervals during the sedation, to collect physiological data and as a routine measure of physiological stability while sedated. A pulse oximeter was attached to one ear to measure the oxygen saturation. Many of the captures took place at night at low ambient temperatures and as thermoregulation can be compromised by sedation, body temperature was closely monitored while the animal was sedated to ensure it did not drop below physiologically accepted levels [29].

### Blood collection and storage

Twenty millilitres of blood was collected from the cephalic vein. Two millilitre aliquots were placed into three separate, two millilitres blood serum separating tubes (Interpath Services PTY Ltd, Heidelberg West, Australia) for later serology analyses. Five millilitres of blood was placed into a tube with ethanol for DNA analyses and one millilitre into a lithium heparin tube for haematology and biochemistry. The remainder of the blood sample was placed on Nobuto strips (10 per animal), which hold 0.1ml of whole blood or 0.04ml of serum (Toyo Roshi Kaisha, Ltd., Tokyo, Japan) and Whatman FTA cards (one per animal) (GE Healthcare UK Limited, Little Chalfont, UK). The advantage of both the Nobuto strips and Whatman FTA cards are they can be stored at room temperature. The FTA cards and Nobuto strips were air dried and stored in paper envelopes.

Blood smears were made in the field from each blood sample collected, then fixed in Diff Kwik fixative at our campsite (Fronine Laboratory Supplies, Riverstone, Australia). Serum tubes stood overnight to separate cells and serum. Serum was decanted into sterile cryovials and held at −18°C until transport to the National Veterinary Institute in Uppsala, Sweden for storage at −80°C until they were tested six to twelve months later.

### External parasites

External parasites were collected during physical exam of the pelage and placed in ethanol for later testing for bacterial pathogens as explained below.

### Haematology

Blood smears were stained with Diff Kwik (eosin and methylene blue) and microscopically examined at 40x and 100x (oil) magnification for cellular abnormalities and haemoparasites.

### Laboratory analyses

Snow leopard serum samples were tested for the presence of antibodies against *Toxoplasma gondii, Coxiella burnetii* and *Leptospira* spp. utilising Enzyme-Linked-Immuno-Assay (ELISA). If positive results were obtained for *Leptospira* spp. the samples were then tested using Microscopic agglutination test (MAT) against a panel of serovars. Next Generation Sequencing (NGS) was used to screen ticks for bacteria (Table 2.). Details of these analyses are outlined below.

**Table 1.** Summary of physiological measurements of snow leopards captured in the Tost Mountains, Mongolia in 2008 to 2015 (mean ±SD). For detailed individual measurements see [27].

Sex	Body weight (kg) (Range)	Heart rate (beats per minute) (Range)	Body temperature (°C) (Range)	Respiratory rate (breaths per minute) (Range)
Male (adult) n = 10	43.1 ± 2.3 (40.7–45)	97.5 ± 10.6 (87–110)	37.3 ± 1.1 (36.9–39.2)	27 ± 4.2 (24–36)
Female (adult) n = 8	36.0 ± 2.9 (32–41.5)	107 ± 5.6 (89–113)	38.4 ± 0.14 (37.2–39.1)	27 ± 4.2 (24–36)
Male (subadult) n = 4	34.8 ± 2.9 (34–39)	98 ± 1.4 (93–101)	37.25 ± 1.1 (36.9–39.3)	21 ± 1.1 (20–22)
Female (subadult) n = 3	28.3 ± 2.8 (25–30)	108 ± 23.3 (100–122)	38. 38.1 ± 0. (37.9–39)	25.5 ± 2.12 (23–50)

**Table 2.** Bacterial genera identified in ticks carried by wild snow leopards in the South Gobi in Mongolia (+ denotes bacteria presence, – denotes bacteria absence).

	Snow leopard ID			
Bacteria genera	M1	M4	M7	F8
*Aeromonas*	+	-	-	-
*Bacillus*	+	+	+	+
*Bacteroides*	+	+	+	-
*Bordetella*	+	-	-	-
*Clostridium*	+	+	+	+
*Corynebacterium*	+	+	-	+
*Coxiella burnetii*	-	+	+	+
*Legionella*	+	+	+	-
*Pandorea*	+	+	+	+
*Rickettsia*	+	+	+	+
*Staphylococcus*	+	+	+	+
*Streptococcus*	+	+	+	+

### Elution of sera from Nobuto strips

Serum was removed from the Nobuto strips following the manufacturer’s instructions (Toyo Roshi Kaisha, Ltd.,Tokyo, Japan). The strips were cut into small pieces and placed in one ml Eppendorf tubes. Serum was eluted and diluted to 10% by placing in 200ul or 400ul phosphate buffer solution (PBS) depending on whether one or both sides of the strip were saturated with blood. After incubating for one hour to allow the serum to enter the PBS, the serum in the Eppendorf tubes was inactivated at 60ºC for one hour, centrifuged and the filter paper removed from the tubes. The resultant sera were stored at −80ºC until analysis.

#### Toxoplasma gondii

Antibodies against *T. gondii* were detected using a commercially available ABNOVA IgG antibody ELISA kit (ABNOVA, Taipei City, Taiwan). As this was a human kit, the methods were modified accordingly. The enzyme conjugate in the kit was changed to an Alkaline phosphatase-conjugated affinipure Goat Anti-Cat IgG (H + L). The methods also called for a 1:40 dilution of the test sample, but as the samples were already diluted 1:10, they only required an additional four-time dilution. All ELISA plates were read on a Multiskan FC microplate photometer, Thermo Scientific machine at 450 nm. Results were calculated by methods outlined in the kit guide.

#### Leptospira *spp*

*Leptospira* antibodies were first identified using a commercially available ELISA kit *Leptospira* IgG (LS-IgG) ELISA kit, (MBS036971, Mybiosource, San Diego, USA). As this was a rodent test kit the conjugating enzyme was replaced with an alkaline phosphatase-conjugated AffiniPur Goat Anti-Cat IgG (H + L) (Jackson ImmunoResearch Laboratories Inc. West Grove, USA) *Leptospira* serovars were then identified using the Microscopic Agglutination Test- MAT [30]. The MAT panel the samples were tested against consisted of *Leptospira* serovars: *L. interrogans* sv Australis, *L. kirschneri* sv Grippotyphosa, *L. interrogans* sv Icterohaemorrhagiae, *L. interrogans* sv Pomona, *L. interrogans* sv Hebdomadis and *L. interrogans* sv Canicola. These serovars were chosen as they had previously been reported to occur in other regions of Mongolia [31,32]. The antigens used were live cultures of referenced strains. All sera that gave a positive reaction at a 1:100 dilution were further titrated in serial two-fold dilutions to titre endpoint that is 50% agglutination. A titre ≥ 100 was therefore deemed positive to exposure to leptospires.

#### Coxiella burnetii

Antibodies against *C. burnetii* were detected using an Innovative Diagnostics Q Fever Indirect Multi-species ELISA kit. The ELISA was performed following the manufacturer’s instructions. The plate results were read at 450 nm, with positive or negative results calculated as described in the kit instructions (Idvet, 310, Grabels. France).

**Ticks** were analysed using Next Generation Sequencing (NGS) [33]. These analyses detect bacterial genera present, as well as endosymbionts or opportunists. Each tick was manually minced with a sterilized scalpel. Three treatments were performed to allow a better harvest of gram-positive bacterial DNA. First, samples were immersed for 1 hour at 37°C in an enzymatic lysis buffer consisting of 20 mM Tris·Cl, pH 8.0, 2 mM sodium EDTA, 1.2% Triton® X-100 and 20 mg/ml of lysozyme as described in the Dneasy™ Tissue Kit handbook. Second, samples were submitted to three freeze-thaw cycles [34]. Finally, 25µl of proteinase K and 200µl of buffer A was added to the sample before an overnight shaking incubation at 56°C. Two hundred µl of this mix was then introduced in a QIAcube (Qiagen®, Hilden) following the manufacturer’s protocol for purification of total DNA from animal tissues. After the extraction step, we performed an Illumina amplicon sequencing following a modified Miseq protocols (16S Metagenomic Sequencing Library Preparation). One extraction of the negative control was added to every batch of 24 samples and two additional negative controls were added to the PCR steps. A mock community sample (HM-783D, BEI resources) containing genomic DNA from 20 bacterial strains at concentrations ranging between 0.6 and 1400pg/µl was also added in triplicate to confirm the reliability of our method. Purified products were quantified using Quant-iT™ PicoGreen® dsDNA Assay Kit following the manufacturer protocol on a fluorimeter (FilterMax F3, Molecular Devices). Quantified products were then pooled in equimolarity and sent to the GIGA Genomics platform (Ulg) for sequencing on an ILLUMINA MiSeq V2 benchtop sequencer [35,36].

## Results

### Health and physiological data

All of the captured snow leopards appeared to be in good physical condition and healthy except for one adult female (ID F9) that weighed 32 kg and that was scored to be in poor to moderate body condition. Her body weight was lower than recorded averages for adult females in this study (Table 1) with bony prominences (scapular spine, ribs and iliac crest) were easily palpable. From her tooth wear, she was determined to be an older animal. Despite the poor to moderate condition, she was negative for antibodies to the tested pathogens and was reproducing. She was later detected on camera trap records together with two cubs and survived for at least 15 months before her collar dropped off and she could no longer be monitored. No ocular lesions or eyelid deformities were present in the animals examined. Oral cavities were clear of ulcers, other visible lesions or gingivitis. Physiological parameters measured from the snow leopards are presented in Table 1. This table combines data reported in Johansson et al. [27] with data from two snow leopards that has not been previously reported.

### Survival of collared snow leopards

Known survival times for snow leopards after the first capture and sample collection was based on how long collars stayed on and how many times they were recaptured. The length of time that the snow leopards were collared ranged from four to 58 months with an average of 20.1 months. Seven of the collared snow leopards had died by the end of this study. Four of these were suspected to have been killed by people when raiding night-time corrals and their collars destroyed. The remaining three animals died of unknown causes

### Haematology

All cellular components in each of the blood smears (red blood cells, white blood cells and platelets) were normal in appearance when viewed under a microscope at 40 x magnification and 100 x (oil immersion). Extra- or intra-cellular haemoparasites were not observed.

### Serology

Four of 20 snow leopards (20%) were seropositive for *T. gondii* (M1, M9, M11 and F7). Five snow leopards (25%) (M4, M10, F1, F7 and F8) were seropositive for *Coxiella burnetii* and four leopards (20%), (F5, M6, M11, M7) were seropositive for *Leptospira* spp. *L. interrogans* sv. Australis was identified in two snow leopards in 2013 (F5, M7) but the other two samples (M6, M11) were not positive in MAT.

### Tick analyses

Four ticks were collected, from four different snow leopards (F8, M4, M7, M1). One hundred and sixteen genera of bacteria were identified in total from the ticks, with the potentially significant zoonotic bacteria listed in Table 2.

## Discussion

All but one of the snow leopards in this study appeared to be clinically healthy and in good physical condition. The only animal that was below optimum body condition was a female that was later observed in camera trap photos together with two cubs. The energetic cost of feeding the cubs could have accounted for her decreased body condition. Ibex numbers in that year were reported to be lower by the local herders due to the previous harsh winter decreasing vegetation availability for the ibex. The apparent reduction of prey numbers, perhaps exacerbated by the need to provide for the cubs may have contributed to her lower body condition.

We collected novel physiological data on heart rate, respiratory rate and body temperature for wild (sedated) snow leopards (Table 2). This reference data is useful for future health assessments of wild populations and also for monitoring captive animals undergoing similar examinations. Due to the low number (18) of wild snow leopards for which physiological data has been reported, it was deemed important to add the data obtained from the two new animals to those published by Johansson et al. [27].

In captive snow leopards, the presence of ocular coloboma (defects affecting many regions of the eye) has been reported on several occasions with the causative aetiology unknown [10,37]. This condition was not observed in any of the 20 wild snow leopards sampled or four additional cubs that were also examined outside of this study (Esson unpub).

We detected antibodies to *T. gondii, L. interrogans* serovar Australis and *C. burnetii* in the snow leopards from the Tost Mountains of Mongolia. These pathogens have not been identified in wild snow leopards before. They can cause serious infections [38–42], even if all snow leopards in this study appeared healthy. Data from radio telemetry showed that the individuals that were seropositive for these pathogens survived for at least 12 to 24 months after collection of samples. This findings show that snow leopards can survive infections by these pathogens without apparent adverse long-term effects. However, we cannot rule out unobserved adverse impacts from these or other pathogens, if the health of animals is reduced for other reasons. It could also be that some animals succumbed to the effects of infection prior to capture and sampling. These pathogens may also have sub-lethal effects on reproduction that are difficult to quantify. Infections in wild animals may be exacerbated by complex co-factors that reduce health. For example, immune suppression due to stressors such as nutritional compromise, wounds from fighting followed by secondary bacterial infection, other traumas and extreme environmental conditions may affect the pathogenicity of disease. An example is canine distemper virus (CDV) causing a fatal outbreak in lions (*Panthera leo*) [2]. Lion deaths were associated with a heavy co-infection of the haemoparasite *Babesia* combined with the immunosuppressive effects of CDV. In that study, there were increased ticks, vectors of *Babesia*, on ungulate prey due to an extreme drought followed by heavy rains [2].

Should the snow leopard populations decrease in the region, there will be a higher risk of inbreeding depression as has been observed in tiger (*Panthera tigris*) populations [43]. An increase in inbreeding depression can have an impact on the immune system response (for example, a decrease of the variability of the major histocompatibility complex genes as seen in the endangered European mink (*Mustela lutreola*) [44,45]. This could impair the snow leopards’ immune system and hence ability to resist infection, therefore, increasing the risk of mortality linked to these pathogens. This is an extinction vortex, where a species is subjected to multiple stressors (associated with poaching, habitat destruction extreme weather conditions), which will accelerate mortality rates because of the impact of a lower resistance of the immune system to disease and a lower adaptive potentiality to each stressor [46,47]. Therefore there is a need for continual, long-term disease surveillance of snow leopards, including necropsies of dead animals to determine disease impacts. If dead snow leopards are found in the future, protocols need to be in place so that appropriate samples can be collected to determine the cause of death. Due to the risk of exposure to highly noxious zoonoses such as anthrax and plague, personnel trained in sample collection along with a necropsy kit complete with personal protection gear for sampling must be used.

The origin of the identified pathogens is unknown. Nor is it known if snow leopards can act as reservoirs of infection or whether they are dead-end hosts or part of a transmission cycle. Rodents, dogs and goats sampled within the study area were positive for exposure to the same pathogens (i.e. *C. burnetii, T. gondii* and *Leptospira* spp.) in concurrent studies in the region (Esson et al. unpublished data). The snow leopards also overlapped with scavengers, such as raptors and foxes at kill sites, allowing for indirect interactions with other potential hosts and the pathogens they shed. These indirect interactions could include sharing environmental resources that are contaminated with pathogens such as water bodies and thereby being parasitised by common water-borne pathogens. Again this warrants long-term studies on this population of snow leopards to elucidate the source(s) of the pathogens identified in the study and other pathogens that may be present that were not tested for.

Felids are the definitive hosts for *T. gondii*, an obligate intracellular protozoan with a complex life cycle [40,41,48]. Broadly, transmission occurs when oocysts are shed in the faeces by the definitive host and then consumed by an intermediate host, which can be a rodent or another mammal [40]. Antibodies to *T. gondii* occurred in 20% of the snow leopards tested, including males and females and across all years of the study. The cub (F8) of F7 was negative to *T. gondii*, suggesting that F7 did not have an active infection during pregnancy or the pathogen did not cross the placenta in this case. *Toxoplasma gondii* can be transmitted horizontally via an intermediate host or vertically from the mother [49]. F7 may also have acquired the infection after giving birth. These results indicate that snow leopards in this region may be acting as a reservoir host for this parasite. *Toxoplasma gondii* was identified in Pallas cats in an amaig (Province) north of the study area in 2005 [50] and has only recently been identified in humans in Mongolia [51]. We could not find any other report or publication about *T. gondii* in other species in Mongolia, so the prevalence of this parasite across the country is unclear. The snow leopards in our study did not appear to be impacted negatively by this parasite, however, negative effects have been observed to occur in other wild felids from infection with *T.gondi*. These include the death of a juvenile bobcat (*Lynx rufus*) from acute toxoplasmosis, and a juvenile cheetah (*Acinonyx jubatus*) a Siberian tiger (*Panthera tigris altaica*) and two lions, which all died from acute disseminated toxoplasmosis [15,50,52–54]. Pallas cats also inhabit the same area as snow leopards, are extremely susceptible to infection with *T. gondii* [50]. This pathogen therefore can have detrimental effects on members of the definitive host family [55] so it would be prudent to continue long-term monitoring of the snow leopards to determine if negative impacts do occur. The snow leopards may also act as a reservoir for this pathogen for contamination by intermediate hosts. Toxoplasmosis is a severe zoonosis, that can cause neurological problems in the foetus and adults of its intermediate hosts and spill-over hosts including humans [38,39]. This could pose a public health risk to the nomadic people of the region.

*Coxiella burnetii*, the aetiological agent of Q fever, is common in Mongolian domestic animals [50] but has not been reported in wildlife in this region. Therefore, the role wildlife plays in transmission or as a reservoir host of this pathogen is unknown. Antibodies to this pathogen were detected in five of 20 snow leopards including both males and females. This is the first time *C. burnetii* has been recorded in this species in the wild. Although very few species of wild felids have been tested for this pathogen, it has been detected in the European wildcat (*Felis silvestris silvestris*) [56,57]. These studies provide an overview of the level of infection in free-roaming felines and highlight their potential zoonotic risk to humans. Fourteen wild caught Pallas’ cats near the Russian-Mongolian border were reported to be negative for *C. burnetii* in 2010 [50]. However, *C. burnetii* has been detected in other felid species in captivity, such as in aclinical lions in a zoo [58]. Felids, including domestic cats (*Felis catus*), have been identified as potential reservoir hosts for the bacteria and a source of infection for humans and other animals [59–61]. The presence of *C. burnetii* antibodies in the snow leopards suggests they may function as a part of the epidemiological cycle of *C. burnetii* just as domestic cats have been reported to do in other regions [62]. Assessment of the role of wild and domestic hosts as potential reservoirs of misdiagnosed zoonoses, such as Q fever by *C. burnetii*, is an important public health issue today both for wildlife conservation and management of disease in the human–livestock–wildlife interface [56,63]. *Coxiella burnetii* can cause abortions, mainly in ruminants, however, its pathogenicity in cats has not been established to date [64]. F8 who was positive for *C. burnetii* was the cub of F7 who was also positive for *C. burnetii* suggesting vertical transmission of the pathogen or common and continual exposure of this pathogen. Continued monitoring of this population is necessary to assess potential effects on reproduction. As DNA was collected from all animals, parentage of future captures can be determined which in turn can reveal possible effects of *C. burnetii* on subsequent reproduction. The snow leopards may have been exposed to *C. burnetii* when preying on domestic goats, ibex or rodents that also inhabit the area. These species also tested positive for exposure to this pathogen in concurrent studies (Esson unpublished). *Coxiella burnetii* is extremely resistant to environmental conditions being able to withstand cold temperatures and UV light so that it could remain viable in the Mongolian environment for extended time-periods [65]. Ruminants are the primary carriers of this pathogen and shed it in their milk, blood, placenta and faeces [58,61]. However, ticks can act as vectors and other mammals such as rodents can also carry *C. burnetii* [57,66,67]. Vaccinating domestic stock would help control transmission among livestock and potential spill over to native ungulates such as ibex and Argali sheep by reducing the deposition of pathogens onto shared grazing regions. Vaccinating against *C. burnetii* would also potentially help mitigate the potential risk of spill-over to people, dogs and other wildlife, including snow leopards. Ticks from three of the snow leopards tested positive for unidentified species of *Coxiella* but were not from those snow leopards that were seropositive for *C. burnetii*. It is possible that the hosts did not have time to develop antibodies prior to sampling during the study. The role of ticks in the transmission of *C. burnetii* is unknown [67] and thus warrants further research.

Four snow leopards tested positive for *Leptospira* antibodies via ELISA, two of which were positive for *L. interrogans* Australis via MAT. Mean agglutination test is considered the ‘gold standard’ for identification of *Leptospira* serovars [68]. *Leptospira interrogans* Australis is considered one of the pathogenic serovars of the genus *Leptospira* [68]. *Leptospira interrogans* Australis has not previously been identified in any Mongolian species so the finding of previous exposure in snow leopards is important from a public health point of view. The identification of this pathogen indicates there is a potential reservoir in the area that the snow leopards and other wildlife, domestic animals and people can be exposed to. Other *Leptospira* serovars than Australis have been reported in other wild felid species, but the effects are still unclear [69]. Two of the snow leopards that were positive for *T. gondii* were also positive for *Leptospira*, showing they have been exposed to multiple pathogens. No typical clinical signs of infection were observed for either of these two individuals (e.g. pyrexia, jaundice), similar to results from captive neotropical felids in Brazil where an ocelot (*Leopardus pardalis*) and a female marguay (*Leopardus wiedii*) tested positive to two different serovars of *Leptospira* without showing clinical signs of disease [69,70]. *Leptospira* spp. are spirochaete bacteria that reside in the kidney tubules, with rodents being a significant reservoir for the bacteria, exhibiting no clinical signs of disease [71], however other mammals including cats have also been reported as reservoirs of the pathogen [72]. *Leptospira interrogans* Hardjo was the most common serovar in cattle and horses reported in two other provinces of Mongolia [32,73] and ten other serovars were identified in dogs in other provinces of Mongolia [31]. Wild and domestic carnivores in Spain with *Leptospira* spp. antibodies commonly had interstitial glomerular nephritis upon necropsy, despite not exhibiting clinical signs of disease [15]. It was suggested that these carnivores were terminal hosts, unable to transmit the disease [15]. Leptospirosis could, therefore, potentially shorten the lifespan of the animals that were positive for *Leptospira* in our study, if sufficient renal damage has occurred. *Leptospira* spp. are readily transmitted to humans and is a zoonosis of global importance [42].

Bacterial genera, both zoonotic and non-zoonotic, were identified in the ticks collected from the snow leopards. The genus of major concern comprised *Bacillus* spp., which can include *B. anthracis* that causes anthrax and *B. piliformis* that causes Tyzzer’s disease. The latter has previously been described in captive snow leopards where it caused fatal infections [12]. Other genera identified were *Coxiella* spp. and *Rickettsia* spp. that include species that cause anaplasmosis and ehrlichiosis, both intracellular bacteria that infect and destroy white blood cells, plus *Staphylococcus* spp. and *Streptococcus* spp [74–76]. For the majority of these pathogens, there are no previous reports of occurrence in snow leopards in the wild or in captive settings. However, based on our results it would be prudent to test for the presence of these bacteria in future health-screening studies on this and other snow leopard populations. In our study, the snow leopards that tested positive for *C. burnetii* antibodies showed no clinical signs of illness and survived for at least 12 to 24 months. The presence of *Coxiella* spp. in ticks may indicate a mechanism of transmission of the pathogen between snow leopards and other hosts as it has in other species [63,67].

### Conclusions

Disease threats to endangered species tend to be overlooked in light of more apparent threats such as habitat destruction and poaching [72]. This study is the first to detect exposure of wild snow leopards to zoonotic pathogens. Potential sources of the three pathogens identified that is *Coxiella burnetii, Toxoplasma gondii* and *Leptospira interrogans* Australis, include snow leopard prey such as wild and domestic ungulates and overlap with scavengers, such as raptors and foxes at kill sites, allowing indirect interactions with other potential hosts and the pathogens they shed. Such potential indirect interactions could include sharing of common resources that are contaminated with pathogens such as water bodies and being parasitized by water-borne pathogens.

There was no evidence of adverse impacts of the zoonotic pathogens on the health and reproduction of the snow leopards in the Tost Mountains. Identification of these pathogens was based on antibody identification from prior exposure and not an active infection. For species where there is limited information, such as the snow leopard, there is a distinct need to continue long-term monitoring of their health to generate comprehensive baseline knowledge of what are normal parameters. Monitoring the survival and condition of animals over time can be achieved through radio-collaring, continual collection and testing of samples as per this study. Only through continued monitoring, including disease surveillance, can we start to understand the threats to this endangered species.
